# Quantification of ^11^C-PIB kinetics in cardiac amyloidosis

**DOI:** 10.1007/s12350-018-1349-x

**Published:** 2018-07-23

**Authors:** Tanja Kero, Jens Sörensen, Gunnar Antoni, Helena Wilking, Kristina Carlson, Ola Vedin, Sara Rosengren, Gerhard Wikström, Mark Lubberink

**Affiliations:** 1 Department of Surgical Science, Uppsala University, Uppsala, Sweden; 2 Department of Medicinal Chemistry, Uppsala University, Uppsala, Sweden; 3 Department of Medical Sciences, Uppsala University, Uppsala, Sweden; 4 Medical Imaging Centre, Uppsala University Hospital, Uppsala, Sweden; 5 Department of Hematology, Uppsala University Hospital, Uppsala, Sweden; 6 Department of Cardiology, Uppsala University Hospital, Uppsala, Sweden; 7 Department of Medical Physics, Uppsala University Hospital, Uppsala, Sweden; 8 PET Center/Medical Imaging Center, Uppsala University Hospital, 75185 Uppsala, Sweden

**Keywords:** Cardiac amyloidosis, ^11^C-PIB, retention index, standardized uptake value, absolute quantification

## Abstract

**Background:**

The purpose of this work was to determine the optimal tracer kinetic model of ^11^C-PIB and to validate the use of the simplified methods retention index (RI) and standardized uptake value (SUV) for quantification of cardiac ^11^C-PIB uptake in amyloidosis.

**Methods and results:**

Single-tissue, reversible and irreversible two-tissue models were fitted to data from seven cardiac amyloidosis patients who underwent ^11^C-PIB PET scans and arterial blood sampling for measurement of blood radioactivity and metabolites. The irreversible two-tissue model (2Tirr) best described cardiac ^11^C-PIB uptake. RI and SUV showed high correlation with the rate of irreversible binding (*K*_i_) from the 2Tirr model (*r*^2 ^=0.95 and *r*^2 ^=0.94). Retrospective data from 10 amyloidosis patients and 5 healthy controls were analyzed using RI, SUV, as well as compartment modelling with a population-average metabolite correction. All measures were higher in amyloidosis patients than in healthy controls (*p*=.001), but with an overlap between groups for *K*_i_.

**Conclusion:**

An irreversible two-tissue model best describes the ^11^C-PIB uptake in cardiac amyloidosis. RI and SUV correlate well with *K*_i_ from the 2Tirr model. RI and SUV discriminate better between amyloidosis patients and controls than *K*_i_ based on population-average metabolite correction.

**Electronic supplementary material:**

The online version of this article (10.1007/s12350-018-1349-x) contains supplementary material, which is available to authorized users.

## Introduction

In amyloidosis, different types of insoluble proteins, amyloid fibrils, are deposited extracellularly in various tissues, leading to progressive organ dysfunction.^1^ Cardiac involvement in amyloidosis is associated with high morbidity and mortality due to arrhythmia, ischemia and progressive heart failure^2^ why a reliable and early diagnosis is important for appropriate management.

The ^11^C-labelled PET tracer Pittsburg compound B (^11^C-PIB) was developed for visualization and quantification of amyloid deposits in the brain in Alzheimer´s disease.^3^ This tracer is also able to visualize amyloid deposits in the heart in patients with both immunoglobulin light-chain (AL) and transthyretin-related (ATTR) amyloidosis^4^,^5^ and a recent study has shown similar results for ^18^F-florbetapir PET.^6^ PET is thus a promising non-invasive tool for specific diagnosis and follow-up after treatment in patients with cardiac amyloidosis.

The clinical utility of the PET examination depends among other things on level of complexity and length of the PET procedure as well as the ability to yield accurate and reproducible results. Retention index (RI) is a simple analysis method, which seems to perform well with amyloid-specific PET tracers as a diagnostic tool for cardiac amyloidosis.^4^,^7^ Standardized uptake value (SUV), SUV ratios and target–to-background ratio (TBR) are other simplified analysis methods, all of which have demonstrated higher values in amyloidosis patients than in controls.^5^,^6^ However, these measures cannot differentiate between amyloid-specific binding and non-specific tracer uptake, tracer in the blood-pool, spill-in from surrounding tissues or radioactive metabolites, which probably explain why also healthy volunteers have had non-zero values. Detecting early amyloidosis and assessing small changes in the amyloid load after therapy or progression of disease might therefore be challenging for RI and other simple analysis models. RI and SUV have yet not been validated against full compartment modelling and metabolite analysis in cardiac amyloidosis.

Therefore, the aims of this study were to determine the optimal tracer kinetic model for analysis of ^11^C-PIB data and to evaluate the performance of two simplified methods, retention index (RI) and standardized uptake value (SUV), in the quantification of cardiac ^11^C-PIB uptake in amyloidosis. Finally, all methods were applied to a previously acquired dataset including both amyloidosis patients and healthy controls to address the ability of each method to discriminate between patients and controls.

## Methods

### Patient Population

Nine patients (mean age 68 years, range 54-78; 7 males) with systemic amyloidosis and heart involvement were included in this prospective study. All patients had immunohistochemistry-confirmed amyloid disease of AL- or ATTR-type. Heart involvement was diagnosed by myocardial biopsy (N=2) or echocardiography (N=6), according to the criteria published by Gertz et al,^8^ whereas in one patient heart involvement was diagnosed by cardiac magnetic resonance imaging.^9^^–^11 Written informed consent was obtained from all subjects and the study was performed with permission from the Regional Board of Medical Ethics in Uppsala and in accordance with the declaration of Helsinki.

One patient with AL-type of amyloidosis died before the ^11^C-PIB PET-scan. Table 1 summarises the patient data for the remaining eight patients.

**Table 1:** Patient data

Subject	Age (years)	Sex	Diagnosis	Cardiac involvement diagnostic method	Duration of disease
1	67	F	TTR hereditary	Fat pad biopsy + echocardiography	2 years, 10 months
2	74	M	TTR senile	Myocardial biopsy	10 months
3	63	M	TTR hereditary	Fat pad biopsy + echocardiography	2 years, 9 months
4	76	M	TTR senile	Fat pad biopsy + echocardiography	5 months
5	78	M	TTR senile	Myocardial biopsy	1 year
6	54	F	AL lambda (myeloma)	Fat pad biopsy + echocardiography	5 months
7	67	M	AL lambda	Fat pad biopsy + echocardiography	2 months
8	70	M	AL lambda	Fat pad biopsy + MRT	5 months

Subject	Treatment	NYHA class	NTpro BNP (ng/L)	Ischemic heart disease	Cardiac medication
1	Ursodeoxycholic acid, Tetracycline, Tafamidis or placebo in study	II	1973	No	Beta blocker, Long-acting nitrate
2	Ursodeoxycholic acid, Tetracycline	II	1291	No	Beta blocker, Long-acting nitrate
3	Ursodeoxycholic acid, Tetracycline	II	962	No	None
4	Ursodeoxycholic acid, Tetracycline	III	7501	Yes	Diuretic, ARB
5	Ursodeoxycholic acid, Tetracycline	III	3306	No	Beta blocker, Diuretic, ACE-inhibitor
6	Bortezomib, Dexamethasone	III	3301	No	Digoxin
7	Bortezomib, Dexamethasone	II	3328	Yes	Beta blocker, Diuretic, ARB
8	Cyclophosphamide, Dexamethasone	III	2627	No	Beta blocker, Diuretic, ACE-inhibitor

*MRI*, Magnetic resonance imaging; *NYHA class*, the New York Heart Association functional classification; *NT pro-BNP*, N-terminal pro b-type natriuretic peptide; *ARB*, Angiotensin receptor blocker; *ACE*, Angiotensin-converting enzyme

### Scanning Protocol

After a respiration-averaged low-dose CT scan, a 35-minute dynamic emission scan of the heart was started simultaneously with intravenous bolus injection of ^11^C-PIB (5 MBq/kg) on a Discovery ST PET/CT (subject 1-6) or Discovery MI scanner (subject 7-8) (GE Healthcare). Recovery was matched in the two scanners based on previous measurements with a NEMA image quality phantom. Imaging was performed in 3D-mode. All appropriate corrections for normalization, dead time, decay, scatter, randoms and attenuation were applied. Images were reconstructed into 31 frames (12×5, 6×10, 4×30, 2×60, 2×120 and 5×300 seconds) using ordered subset expectation maximization (OSEM) with 2 iterations and 21 subsets (Discovery ST) or time-of-flight OSEM with 3 iterations and 16 subsets (Discovery MI) and a 5 mm gaussian post-filter. Images consisted of 128×128 voxels, with dimensions of 2.34×2.34×3.27 mm (Discovery ST) and 2.34×2.34×2.79 mm (Discovery MI), and a spatial resolution of approximately 7 mm.

### Blood Sampling and Input Functions

All subjects received a radial artery catheter for arterial blood sampling during the dynamic PET-scan. Discrete blood samples (5 mL) were drawn manually at circa 2.5, 5, 10, 15, 20, 25 and 35 minutes post injection. For each sample, activity concentrations in whole blood and plasma were determined. The percentage of intact ^11^C-PIB in plasma was determined by HPLC analysis using UV- and radio detection: an 1.8 mL sample was injected onto a semi-preparative HPLC column (Genesis C18, 7 µm, 250×10 mm, Phenomenex) equipped with a guard column (C18 SecurityGuard, 10×10 mm, Phenomenex). The column was eluted at a flow rate of 6 mL·min with acetonitrile-50 mM ammonium acetate pH 5.3 (55:45, v/v). The outlet from the detector was connected to a switching valve on the arm of the liquid handler to enable automatic fraction collection. Three fractions were collected, the first two containing the metabolites and the third containing the unmetabolized parent compound, and the radioactivity in each fraction was measured by a well-type scintillation counter.

Regions of interest were placed in the aorta in 10 consecutive transaxial planes and then combined into a volume of interest (VOI). A second VOI was placed over the right ventricular cavity. These VOIs were transferred to the dynamic image sequence to obtain the left and right ventricular time-activity curves (TACs). Input functions were calculated by multiplication of the left-ventricular TAC with a single exponential fit to the measured plasma—whole blood ratios and a sigmoid fit to the fraction of unmetabolized ^11^C-PIB in plasma.

### Data Analysis

#### Volumes of interest

The dynamic ^11^C-PIB scan was analyzed using Carimas software (version 2.63) developed at Turku PET Centre in Finland (www.turkupetcentre.fi/carimas/). Myocardial segment VOIs were semi-automatically drawn over the left ventricle according to the 17-segment model of the American Heart Association^12^ and segmental TACs were extracted.

#### Tracer kinetic modelling

Whole-myocardium and segment TACs were fitted to a single-tissue compartment model (1T), an irreversible two-tissue compartment model (2Tirr), as well as a reversible two-tissue compartment model (2Trev) and two variations of this last model where the non-specific distribution volume *K*_1_/*k*_2_ or both *K*_1_/*k*_2_ and *k*_4_ were fixed to their whole-myocardium values, respectively. In addition, a dual-input single-tissue model (1T-1T), with parallel compartments for ^11^C-PIB and radioactive metabolites, was evaluated, accounting for the possibility that radioactive metabolites of ^11^C-PIB enter myocardial tissue. Fitted corrections for spill-over from left and right ventricular cavities were included in all models and fits were performed using non-linear regression in in-house developed software in Matlab. Outcome measure for the 1T model was the volume of distribution *V*_T_ (= *K*_1_/*k*_2_), for the 2T models *V*_T_ = *K*_1_/*k*_2_ (1+*k*_3_/*k*_4_) and the binding potential BP_ND_ were evaluated, whereas for the irreversible models the net influx rate *K*_i_ (= *K*_1_*k*_3_/(*k*_2_+*k*_3_)) was used.

To exclude unreliable fits, fits with outcome parameters with standard errors larger than 25% were discarded. The best fit was determined using the Akaike information criterion (AIC)^13^ and Akaike weights.^14^ The AIC was defined according to Eq. 1:

1 $$ {\text{AIC}} = {\text{N}} \times \ln \left( {\text{WSSE}} \right) + 2 \times p $$

in which N is the number of frames (31 in the present study), WSSE is the weighted squared sum of residual fit errors and *p* is the total number of parameters for each model. The Akaike weights were defined according to Eq. 2:

2 $$ {\text{AIC}}w_{i} = \frac{{{ \exp }\left( { - \frac{1}{2}\Delta_{i} } \right)}}{{\mathop \sum \nolimits_{i = 1}^{m} { \exp }\left( { - \frac{1}{2}\Delta_{i} } \right)}} $$

where

3 $$ \Delta_{i} = {\text{AIC}}_{i} - {\text{AIC}}_{\hbox{min} } $$

Here, AIC_i_ is the AIC value for each individual model, and AIC_min_ is the lowest AIC value across the different models.

#### Simplified methods

RI_15-25_ was calculated as the mean ^11^C-PIB radioactivity concentration between 15 and 25 minutes after injection divided by the integral of the arterial whole blood TAC between 0 and 20 minutes, as described in detail previously.^7^ SUV_15-25_ was calculated as the mean ^11^C-PIB radioactivity concentration between 15 and 25 minutes after injection normalized to the injected dose divided by patient weight. This time frame was chosen as it was used when calculating the ^11^C-PIB RI in our previous work.^4^ Correlations between RI, SUV and the outcome parameter of the preferred model were assessed using linear regression. In addition, RI and SUV were calculated between 10 and 20, 20 and 30, 25 and 35 and between 10 and 30 minutes post injection to assess time-depending variations in correlations with fully quantitative data.

#### Population-averaged metabolite correction

A population-averaged correction for plasma/whole blood ratios and parent fractions was calculated using the data from the subjects who completed the study. Tracer kinetic modelling was repeated using input functions based on this correction and correlation between the outcome measures was assessed using linear regression analysis and intraclass correlation coefficient (ICC). In addition, correlation between RI, SUV and outcome parameters of tracer kinetic analysis based on population-averaged blood data was assessed using linear regression.

#### Retrospective data

Retrospective data from 10 amyloidosis patients with heart involvement and 5 healthy controls that has been described in detail previously,^4^ were analyzed using the population-averaged metabolite correction and the optimal tracer kinetic model. RI and SUV between 15 and 25 minutes were also calculated for this retrospective dataset. Correlations between RI, SUV and the outcome parameter of the preferred model were assessed using linear regression. Differences in tracer kinetic model outcome, SUV and RI between amyloidosis patients and healthy controls were assessed using Mann–Whitney *U* test. Further, instead of using Cohen´s d, which measures the difference between the means of two groups in terms of their pooled SD, we measured the discriminative power as the difference between the lowest value of the outcome measure, RI and SUV in patients and the mean respective value in controls in terms of the SD of the control group. This method was chosen because of the much skewed, non-normal, distribution of and large spread in values in the patients.

## Results

**Figure 1:**
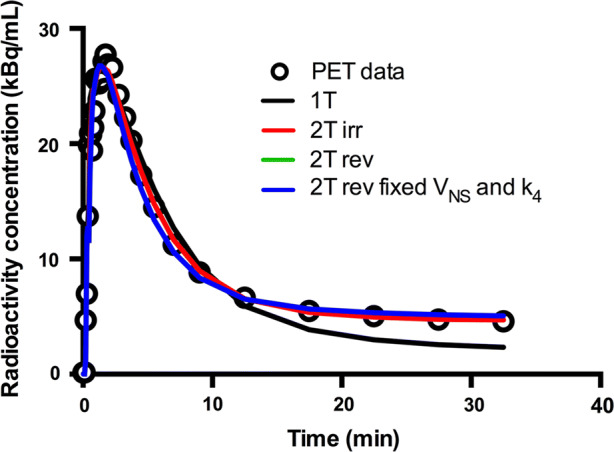
^11^C-PIB time-activity curve of a myocardial segment in a patient with cardiac amyloidosis. Black, red, green and blue lines represent best fits according to single-tissue (1T), irreversible two-tissue (2Tirr) and to two different reversible two-tissue compartment models (2Trev model and 2Trev model with fixed Vns and fixed k4). 2Tirr model fit is superimposed over 2Trev model fit

The analysis of the percentage of intact ^11^C-PIB in plasma failed in one patient (subject number 6 in Table 1) and the data from this patient were excluded from further analysis. An example of a myocardial TAC from a typical patient together with corresponding fits is shown in Figure 1.

### Tracer Kinetic Modelling

Based on AIC, the 2Trev model was preferred in 45 out of 119 VOIs followed by the 2Tirr model (38/119) and the 2Trev model with fixed *K*_1_/*k*_2_ and fixed *k*_4_ (28/119). The 2Trev model with fixed *K*_1_/*k*2 was preferred in only 7 out of 119 VOIs, the 1T-1T model in 1/119 and 1T-model in 0/119. Mean Akaike weights for the three preferred models were 0.41, 0.22 and 0.14, respectively. However, the 2Trev model was unable to provide robust estimates of either *V*_T_ or BP_ND_, with standard errors frequently larger than the parameters themselves. To a lesser extent, this was also the case for the 2Trev models with fixed *K*_1_/*k*_2_ or fixed *K*_1_/*k*_2_ and *k*_4_. The 2Tirr model, however, provided robust parameter estimates in all VOIs and was therefore chosen as the preferable model. When omitting the 2Trev model from the Akaike analysis, the 2Tirr model was preferred in 50 out of 119 VOIs [followed by the 2Trev models with fixed *K*_1_/*k*_2_ and fixed *k*_4_ (31/119) and fixed *K*_1_/*k*_2_ (23/119)] and the Akaike weight increased to 0.39 for the 2Tirr model. The global mean value of the total net influx rate, *K*_i_, using the 2Tirr model, was 0.043 (range 0.014-0.125) mL·cm^3^·minute.

### Simplified Methods

**Figure 2:**
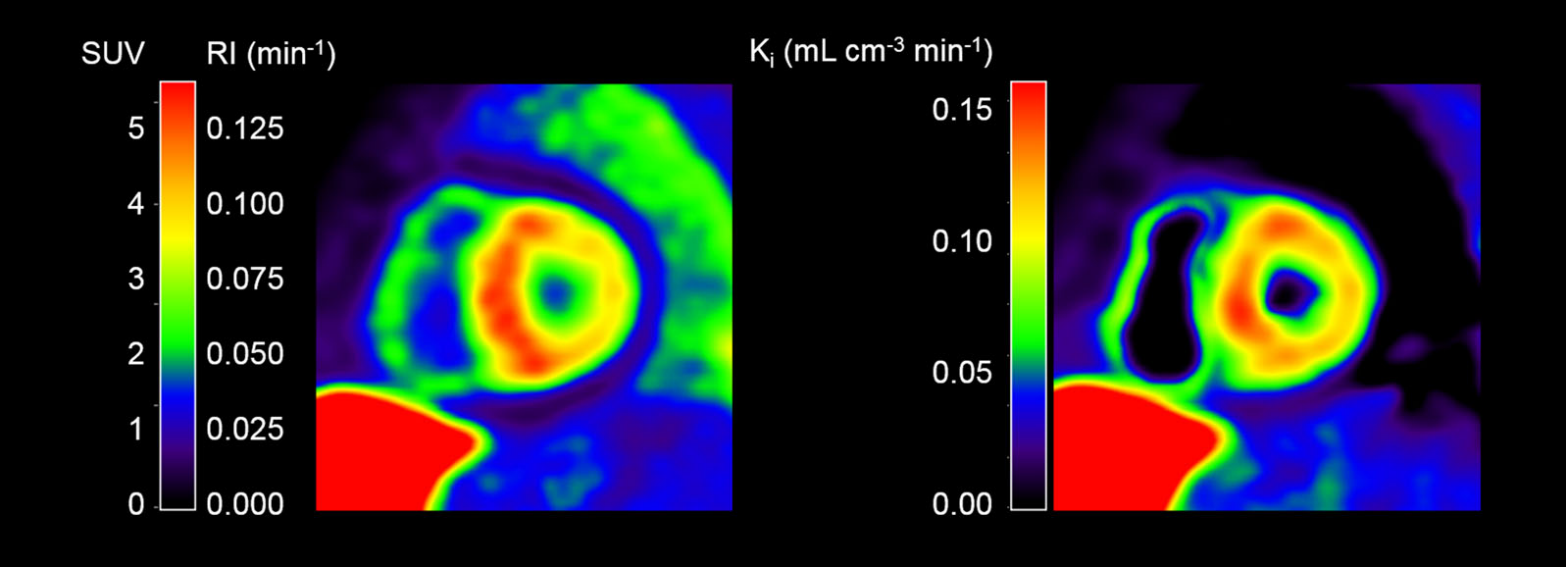
Cardiac short axis ^11^C-PIB images from a patient with AL-amyloidosis. Left: SUV/RI image. Right: Net influx rate *K*_i_ image calculated using a basis function implementation of the 2Tirr model

**Figure 3:**
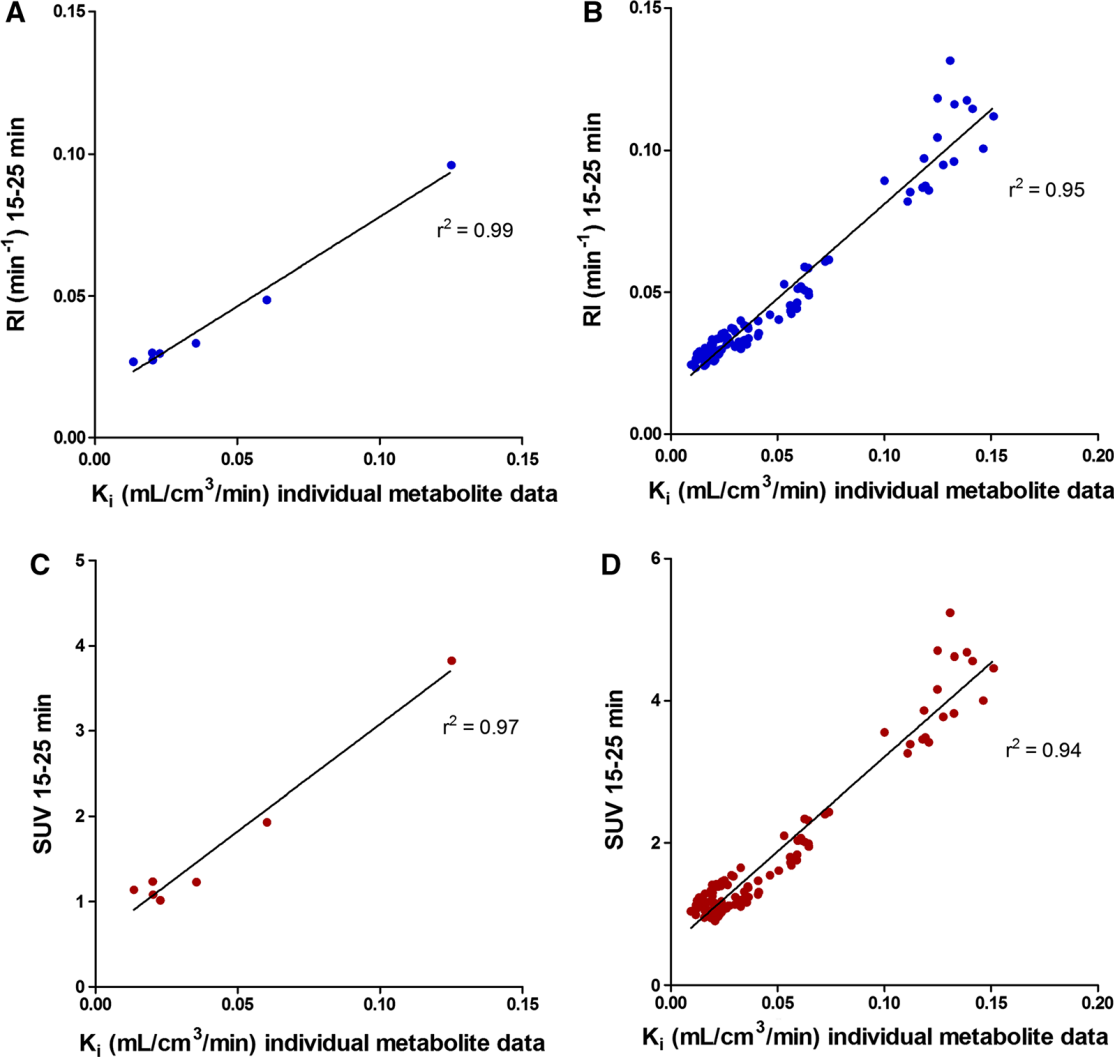
Global and segmental RI_15-25_ (**A** and **B**) respective SUV_15-25_ (**C** and **D**) as a function of *K*_i_ from 2Tirr model

Global mean RI_15-25_ was 0.042 (range 0.027-0.096) min^−1^ and global mean SUV_15-25_ was 1.6 (range 1.0-3.8). Figure 2 shows parametric SUV/RI- and *K*_i_-images from one patient. Figure 3 shows the relationships of global and segmental RI_15-25_ respective SUV_15-25_ with the net influx rate (*K*_i_) from the 2Tirr model. There was a clear correlation of global and segmental RI_15-25_ with *K*_i_ (*r*^2^ = 0.99 and *r*^2^ = 0.95) and of global and segmental SUV_15-25_ with *K*_i_ (*r*^2^ = 0.97 and *r*^2^ = 0.94). However, it was also clear that the relationships of RI_15-25_ and SUV_15-25_ with *K*_i_ varied between the subjects as shown in Table 2. Furthermore, correlations between RI, SUV and *K*_i_ varied with time, as shown in Table 3.

**Table 2:** Parameters of correlation between segmental *K*_i_ 2Tirr and RI_15-25_ and SUV_15-25_ for each subject, mean values of all individual parameters (mean) and parameters of correlation for the whole dataset (total)

	Ki 2T irr vs RI		Ki 2T irr vs SUV	
	*r*^2^	Slope (95% CI)	*r*^2^	Slope (95% CI)
1	0.93	0.77 (0.65–0.88)	0.93	31.82 (27.12–36.53)
2	0.90	0.85 (0.69–1.00)	0.90	35.77 (29.23–42.30)
3	0.71	0.66 (0.43–0.89)	0.71	24.22 (15.72–32.72)
4	0.63	0.64 (0.37–0.92)	0.63	25.15 (14.43–35.88)
5	0.94	0.74 (0.64–0.84)	0.94	25.32 (21.85–28.79)
7	0.40	0.70 (0.23–1.18)	0.40	27.96 (9.16–46.77)
8	0.61	0.83 (0.47–1.19)	0.61	32.92 (18.54–47.29)
Mean	0.73	0.74	0.73	29.02
Total	0.95	0.66 (0.64–0.69)	0.94	26.58 (25.32–27.89)

**Table 3:** Correlation between segmental *K*_i_ 2Tirr and RI and SUV calculated from different time frames

	10–20 minutes	15–25 minutes	20–30 minutes	25–35 minutes	10–30 minutes
*r*^2^ RI vs Ki	0.94	0.95	0.97	0.97	0.96
*r*^2^ SUV vs Ki	0.94	0.94	0.95	0.95	0.81

### Population-Averaged Metabolite Correction

**Figure 4:**
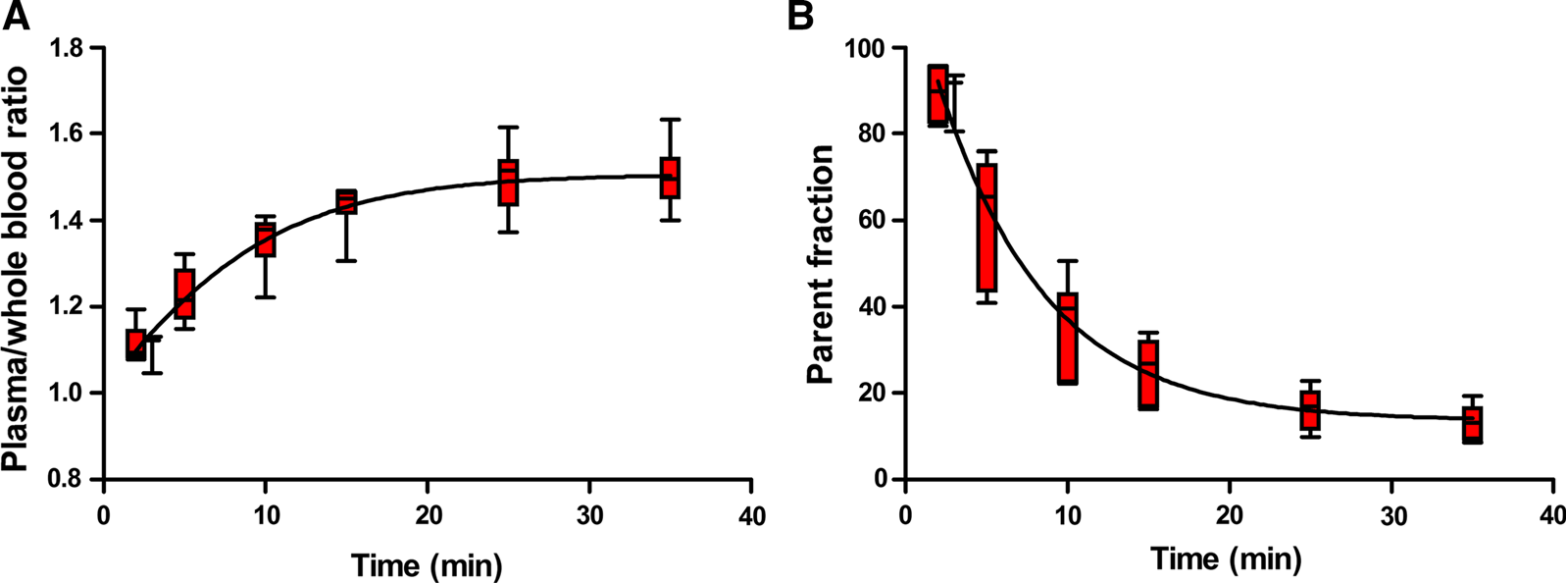
Plasma/whole blood concentration ratio (**A**) and parent fraction in arterial plasma (**B**) as a function of time. Whiskers show min and max values

**Figure 5:**
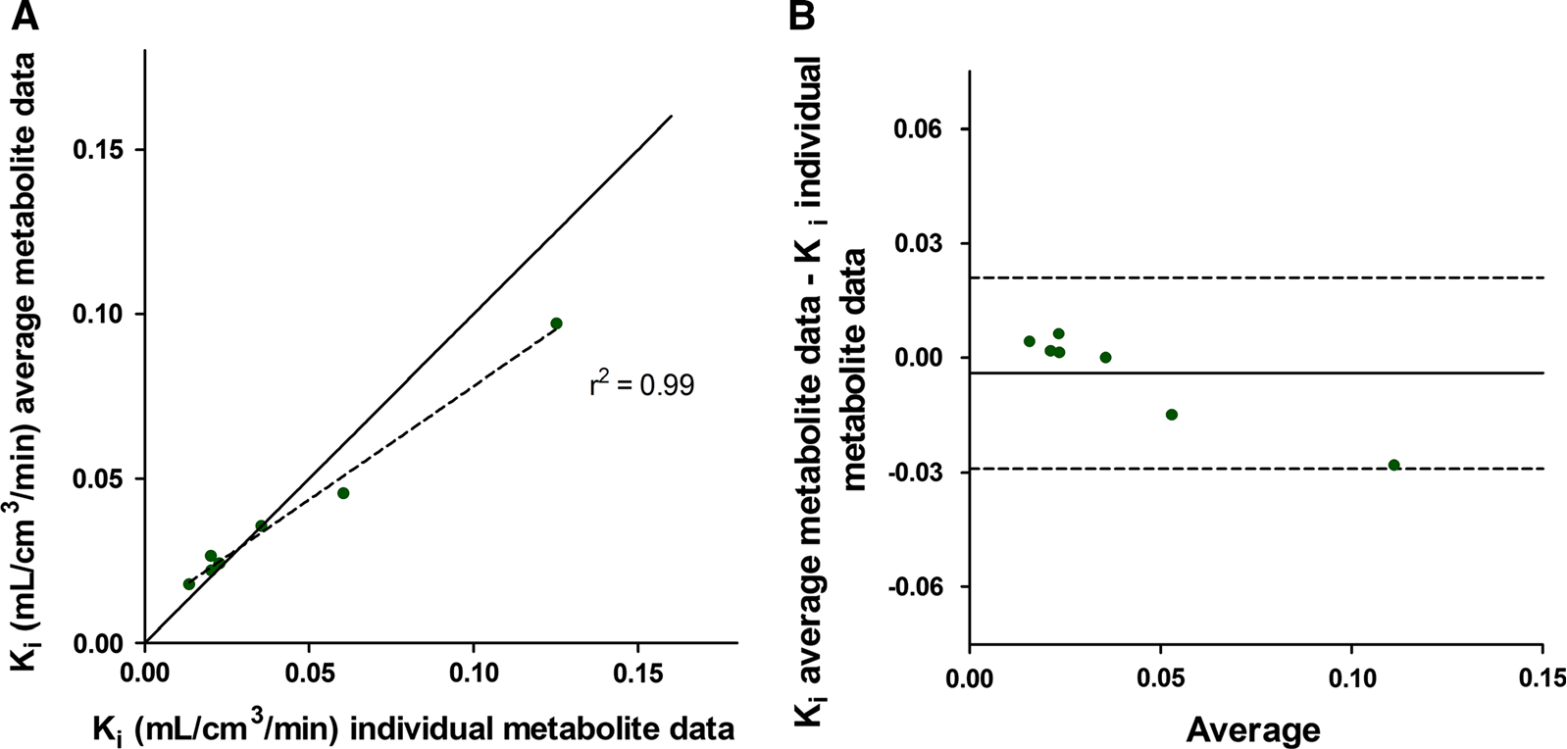
Correlation (**A**) and Bland-Alman plot (**B**) of global mean K_i_ calculated using individual metabolite corrections (horizontal axis) and *K*_i_ calculated using population-averaged metabolite correction (vertical axis). Line of identity is shown as a solid line and regression line as a dashed line (**A**). The solid line in **B** indicates the mean difference (bias), whereas the dashed lines show the limits of agreement. Bias (limits of agreement) are − 0.004 (− 0.029 to 0.021)

Figure 4 shows plasma/whole blood ratios and mean parent ^11^C-PIB fractions as a function of time. There was a rapid metabolism of ^11^C-PIB resulting in a large fraction of labelled metabolites towards the end of the scan (over 80% of the measured radioactivity at 35 minutes), with a substantial variation between subjects. Global mean *K*_i_ was 0.038 (range 0.018-0.097) mL·cm^3^·minute using population-averaged metabolite corrections. Figure 5 shows a scatter-plot of global mean *K*_i_ calculated with individual and with population-averaged metabolite corrections. Correlation (*r*^2^ = 0.99) and agreement (ICC = 0.97) were high, although for two patients *K*_i_ based on population-averaged metabolite correction resulted in lower values than *K*_i_ based on individual metabolite correction.

#### Retrospective data

**Figure 6:**
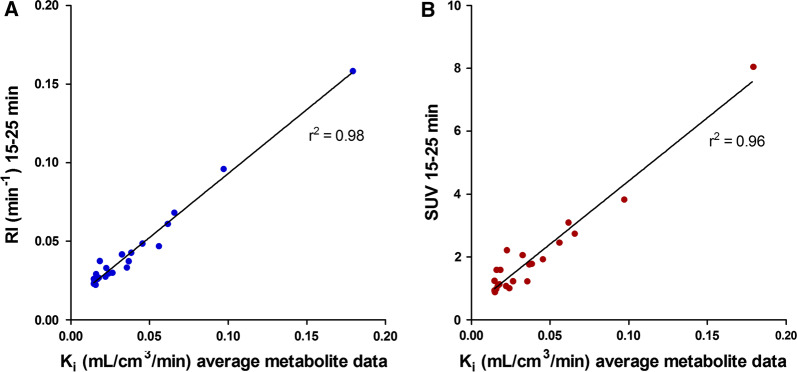
RI_15-25_ (**A**) and SUV_15-25_ (**B**) from retrospective data including amyloidosis patients and healthy controls as a function of *K*_i_ 2Tirr model using population-averaged metabolite correction

**Figure 7:**
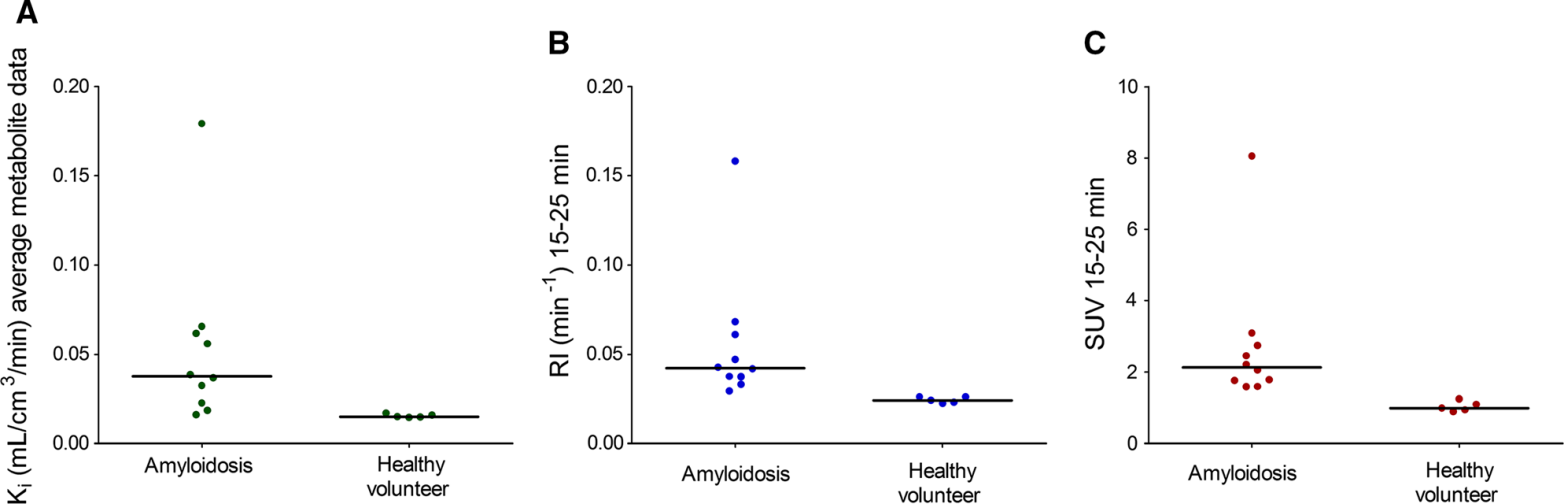
Scatter dot plot diagrams of the myocardial global mean *K*_i_ based on population-average metabolite correction (**A**), RI_15-25_ (**B**) and SUV_15-25_ (**C**) in amyloidosis patients and in healthy controls. Lines indicates median values

When retrospective data from 10 amyloidosis patients and 5 healthy controls were analyzed with the population-averaged metabolite correction and the 2Tirr model, the global mean K_i_ in amyloidosis patients was 0.053 (range 0.016-0.179) mL·cm^3^·minute, compared with 0.015 (range 0.015-0.017) mL·cm^3^·minute in healthy controls. The correlations of global mean RI_15-25_ and SUV_15-25_ with *K*_i_ were high (*r*^2^ = 0.98 and *r*^2^ = 0.96, respectively) as shown in Figure 6. There was a significant difference in *K*_i_ between amyloidosis patients and healthy controls (*p* = 0.001), although there was an overlap between the lowest *K*_i_ in amyloidosis patients and the highest *K*_i_ in controls (Figure 7). For comparison, global mean RI_15-25_ was 0.056 (range 0.029-0.158) min^−1^ in amyloidosis patients and 0.024 (range 0.022-0.026) min^−1^ in controls (*p* = 0.001) and global mean SUV_15-25_ was 2.7 (range 1.6-8.0) in amyloidosis patients and 1.0 (range 0.9-1.2) in controls (*p* = 0.001). Using a modified effect size measure the difference between patients and healthy volunteers was greater for RI and SUV than for *K*_i_ (2.99 SD between lowest RI in amyloidosis patients and mean RI in controls, whereas the respective effect size measures for SUV and *K*_i_ were 2.54 SD and 1.11 SD).

## Discussion

In the present study, the optimal tracer model for kinetic analysis of ^11^C-PIB was determined and the performance of two simpler measures, RI and SUV, in the quantification of cardiac ^11^C-PIB uptake in amyloidosis was evaluated. Finally, all methods were applied to a previously acquired dataset including both amyloidosis patients and healthy volunteers to address the ability of each method to discriminate between patients and controls.

### Tracer Kinetic Modelling

The exact mechanism and kinetics of ^11^C-PIB binding to amyloid are not known. For fully quantitative brain studies using compartment modelling, reversible two-tissue models best described the ^11^C-PIB kinetics,^15^,^16^ although there is some discussion on whether an irreversible model is also appropriate for the scan durations typically used in PET studies.^16^ In our study the reversible two-tissue models were unable to provide robust estimates of the outcome parameters, which the irreversible two-tissue model did. Omitting the 2Trev-model Akaike criteria and Akaike weights indicated that the 2Tirr model was the preferred model to describe myocardial ^11^C-PIB kinetics. A longer scan-time, as was used in the kinetic brain studies, could hypothetically provide more robust fits for reversible models also in cardiac studies. On the other hand, the reversible two-tissue models are more complex as they contain more parameters that have to be estimated, introducing more uncertainty. Moreover, towards the end of the dynamic ^11^C-PIB scans the activity in the myocardium was very low, which is why increasing the scan-time probably would not yield different modelling results in cardiac studies.

### Simplified Methods

Since fully quantitative studies with arterial blood sampling and metabolite analysis are not feasible in routine clinical practice, simplified analysis methods are needed. The first studies on ^11^C-PIB-imaging of brain β-amyloid in Alzheimer´s disease used SUV as a measure of ^11^C-PIB uptake^3^ and subsequent brain studies have used simplified reference tissue models and a target-to-reference ratio in a late time interval.^17^^–^19 Due to the propensity of amyloidosis affecting multiple organs, reference tissue models are less suitable for quantification in cardiac amyloidosis. The simplified measures RI and SUV seem to perform well with amyloid-specific PET tracers in cardiac amyloidosis studies.^4^,^6^ SUV ratios and target to background ratio (TBR) are other simple analysis models that have been used in cardiac amyloidosis studies,^5^,^6^ but were not evaluated in our study.

Our study showed a high correlation of RI and SUV with the total net influx rate, *K*_i_, using the 2Tirr model (*r*^2^ = 0.99 and *r*^2^ = 0.97 for global values and *r*^2^ = 0.95 and *r*^2^ = 0.94 for segmental values respectively), with lower within-patient correlation for segmental values in most patients and with substantial variation between individuals as shown in Table 2. This can most probably be explained by variations in the metabolism of ^11^C-PIB between patients, although technical challenges in metabolite analysis may also influence the results. Furthermore, the correlations between *K*_i_ and the simplified measures varied when RI and SUV were calculated from different time frames. In the present study, RI and SUV were based on uptake between 15 and 25 minutes post injection. In an earlier study we showed that the difference in mean RI between amyloidosis patients and healthy subjects was greater at an early time frame (10-20 minutes) compared to a late time frame (15-25 minutes).^7^ However, when RI and SUV were calculated using uptake from 10 to 20 minutes post injection in the present study, the correlations with K_i_ were slightly lower (*r*^2^ = 0.94 and *r*^2^ = 0.92, respectively). Both simplified measures correlated better with *K*_i_ when calculated at later time frames, as shown in Table 3.

### Population-Averaged Metabolite Correction

In agreement with ^11^C-PIB brain studies, the fraction of labelled metabolites was large towards the end of the scan^15^,^16^ and therefore a metabolite correction is needed for accurate quantification of ^11^C-PIB. A population-averaged metabolite correction could make quantification of ^11^C-PIB possible without arterial blood sampling. *K*_i_ was not significantly different when using the population-averaged metabolite correction [global mean *K*_i_ 0.038 (range 0.018-0.097) mL·cm^3^·minute vs 0.043 (range 0.014-0.0125) mL·cm^3^·minute; *p* = 0.92) and correlation (*r*^2^ = 0.99) and agreement (ICC = 0.97) were high between *K*_i_ based on population-average metabolite data and *K*_i_ based on individual metabolite data. However, for two patients with faster metabolism, *K*_i_ resulted in lower values when using the population-averaged metabolite correction, clearly demonstrated in Figure 5. There was a substantial variation in the fraction of labelled metabolites of ^11^C-PIB between subjects, as shown in Figure 4, and a population-averaged metabolite correction could therefore result in inaccurate quantitative results for some subjects. When assessing changes within a single patient, for instance before and after treatment, quantification using population-averaged metabolite corrections may be considered, assuming that the intervention does not change the metabolism of PIB. However, this assumption should also be tested as both disease progression and intervention may affect organ function and thus metabolism and confounders.

### Retrospective Data

Using retrospective data from 10 amyloidosis patients and 5 healthy controls, the 2Tirr model with population-averaged metabolite correction resulted in a significant difference in *K*_i_ between patients and controls (*p* = 0.001), although there was an overlap between the lowest *K*_i_ in amyloidosis patients and the highest *K*_i_ in controls. As has been shown before, both RI and SUV values were also significantly higher in patients than in controls.^4^,^6^ Furthermore, in our retrospective data, there was no overlap in RI or SUV values between patients and controls and using a modified effect size measure the difference between patients and healthy volunteers was greater for RI and SUV than for *K*_i_, suggesting that both simplified measures discriminated better between cardiac amyloidosis patients and healthy subjects than the 2Tirr model when based on population-averaged metabolite corrections. Individual metabolite corrections could maybe give other results with better separation of *K*_i_ between patients and controls, but this would not be feasible to use in clinical routine.

### Limitations

Fully quantitative PET studies with arterial sampling and metabolite analysis are technically demanding and prone to errors. Due to the technically demanding and costly procedure the sample size was relatively small. Metabolite analysis of ^11^C-PIB is challenging and the substantial variation in the fraction of labelled metabolites of ^11^C-PIB between subjects could therefore be a result of either technical difficulties in the metabolite analysis or true individual variations in metabolism. However, extensive quality control was applied during the metabolite analysis, measuring recovery in each step, and no systematic errors were found. In one subject, however, the metabolite analysis failed due to technical reasons and the data from this subject was excluded.

Due to the small sample size comparison of metabolism of ^11^C-PIB and *K*_i_ values between subjects with AL- and ATTR-type of amyloidosis could not be done. Furthermore, no healthy volunteers participated in the study, and hence it is not certain that population-average metabolite corrections based on patients with amyloidosis can be used for subjects without amyloidosis.

Heart involvement of amyloidosis was diagnosed by myocardial biopsy in two subjects only and with echocardiography (N = 6) or by cardiac magnetic resonance imaging (N = 1) in the others. For the retrospective data, cardiac involvement of amyloidosis was based on endomyocardial biopsy in 5 subjects, whereas echocardiographic criteria were used for the remaining 5 patients. The controls were considered healthy based on medical history. In the retrospective analysis one patient had lower *K*_i_ than the highest *K*_i_ in controls; this subject had TTR-type of amyloidosis and cardiac involvement was confirmed by endomyocardial biopsy.

## New Knowledge Gained

Until now, the optimal quantitative measure of cardiac amyloid load was not known for patients with cardiac amyloidosis. We have determined the optimal tracer kinetic model of myocardial ^11^C-PIB uptake. An irreversible two-tissue model best described the ^11^C-PIB uptake in cardiac amyloidosis. Simplified measures (RI and SUV) correlate well with the net influx rate, *K*_i_, from the 2Tirr-model.

## Conclusion

An irreversible two-tissue model best describes the ^11^C-PIB uptake in cardiac amyloidosis. RI and SUV showed high correlation with quantitative results from this kinetic model, using either individual or population-average metabolite data. However, RI and SUV are more feasible for use in clinical routine and also showed better discrimination between amyloidosis patients and controls than *K*_i_ based on population-average metabolite correction. Therefore, RI and SUV are preferred in clinical diagnosis of cardiac amyloidosis.

## Electronic supplementary material

Below is the link to the electronic supplementary material.

Supplementary material 1 (PPTX 6050 kb)

## Abbreviations

^11^C-PIB: ^11^C-labeled PET tracer Pittsburg compound B
RI: Retention index
SUV: Standardized uptake value
2TIRR: Irreversible two-tissue model
K_i_: Net influx rate
AL: Immunoglobulin light-chain amyloidosis
ATTR: Transthyretin-related amyloidosis
TBR: Target to background ratio
AIC: Akaike information criterion
