# Supplementary material for: Quantification of 11C-PIB kinetics in cardiac amyloidosis
Source: J Nucl Cardiol. 2018 Jul 23;27(3):774–84. doi: 10.1007/s12350-018-1349-x (PMC7326793; doi:10.1007/s12350-018-1349-x)
Supplement: Supplementary file 1 — Supplementary material 1 (PPTX 6050 kb) [file 12350_2018_1349_MOESM1_ESM.pptx]

## Slide 1
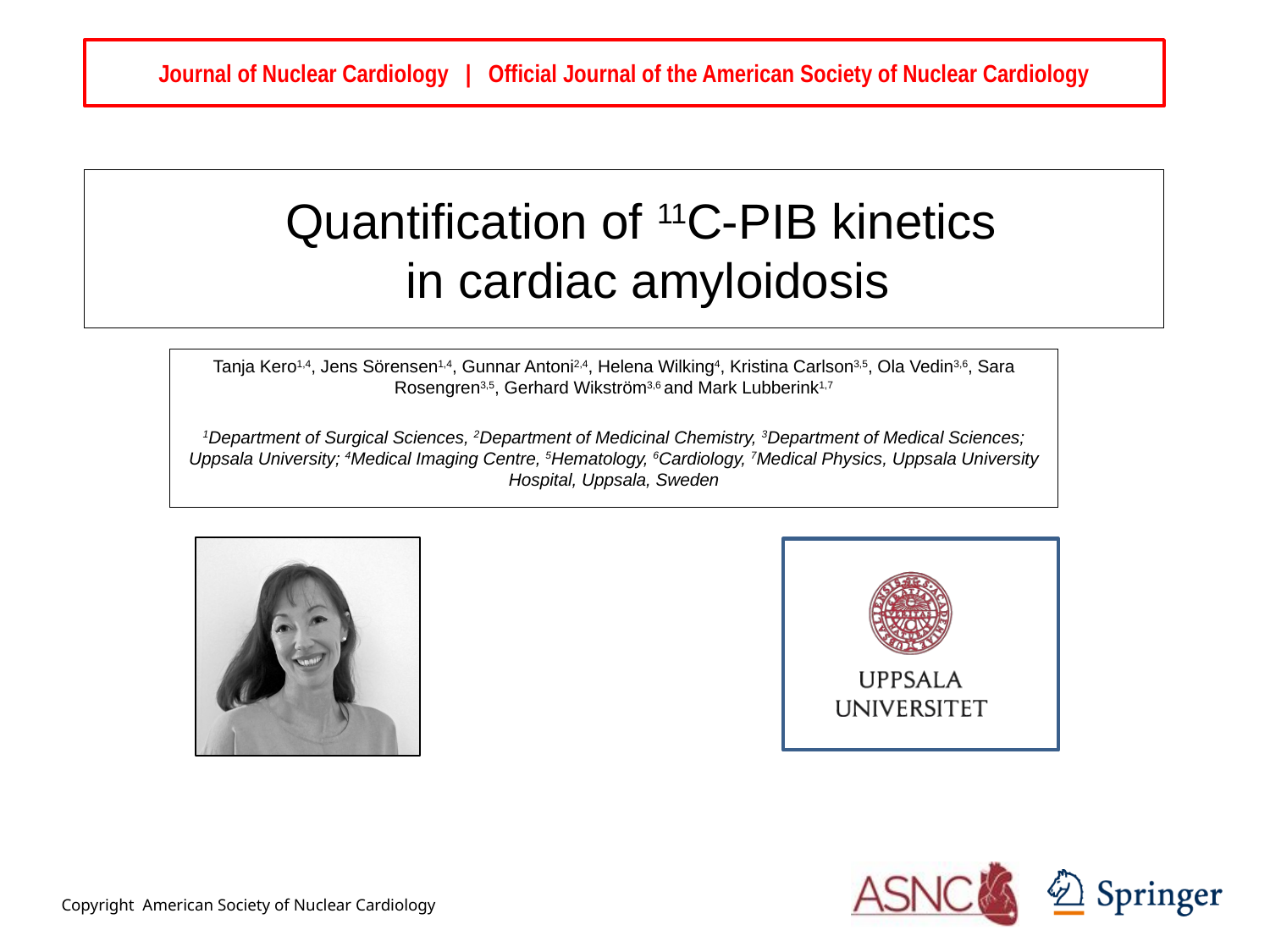

Journal of Nuclear Cardiology | Official Journal of the American Society of Nuclear Cardiology
# Quantification of 11C-PIB kinetics in cardiac amyloidosis
Tanja Kero1,4, Jens Sörensen1,4, Gunnar Antoni2,4, Helena Wilking4, Kristina Carlson3,5, Ola Vedin3,6, Sara Rosengren3,5, Gerhard Wikström3,6 and Mark Lubberink1,7
1Department of Surgical Sciences, 2Department of Medicinal Chemistry, 3Department of Medical Sciences; Uppsala University; 4Medical Imaging Centre, 5Hematology, 6Cardiology, 7Medical Physics, Uppsala University Hospital, Uppsala, Sweden
Copyright American Society of Nuclear Cardiology

## Slide 2
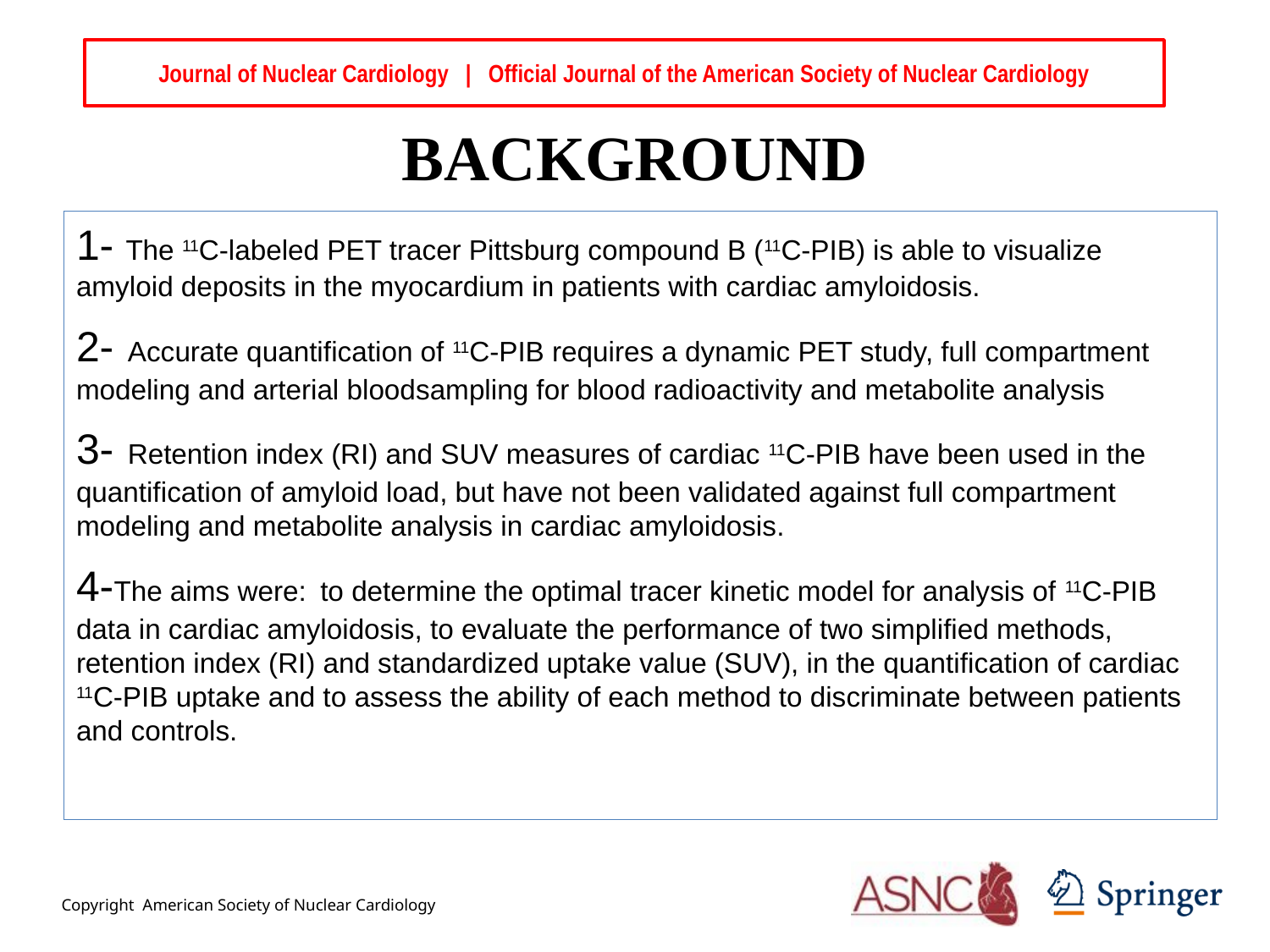

Journal of Nuclear Cardiology | Official Journal of the American Society of Nuclear Cardiology
# BACKGROUND
1- The 11C-labeled PET tracer Pittsburg compound B (11C-PIB) is able to visualize amyloid deposits in the myocardium in patients with cardiac amyloidosis.
2- Accurate quantification of 11C-PIB requires a dynamic PET study, full compartment modeling and arterial bloodsampling for blood radioactivity and metabolite analysis
3- Retention index (RI) and SUV measures of cardiac 11C-PIB have been used in the quantification of amyloid load, but have not been validated against full compartment modeling and metabolite analysis in cardiac amyloidosis.
4-The aims were: to determine the optimal tracer kinetic model for analysis of 11C-PIB data in cardiac amyloidosis, to evaluate the performance of two simplified methods, retention index (RI) and standardized uptake value (SUV), in the quantification of cardiac 11C-PIB uptake and to assess the ability of each method to discriminate between patients and controls.
Copyright American Society of Nuclear Cardiology

## Slide 3
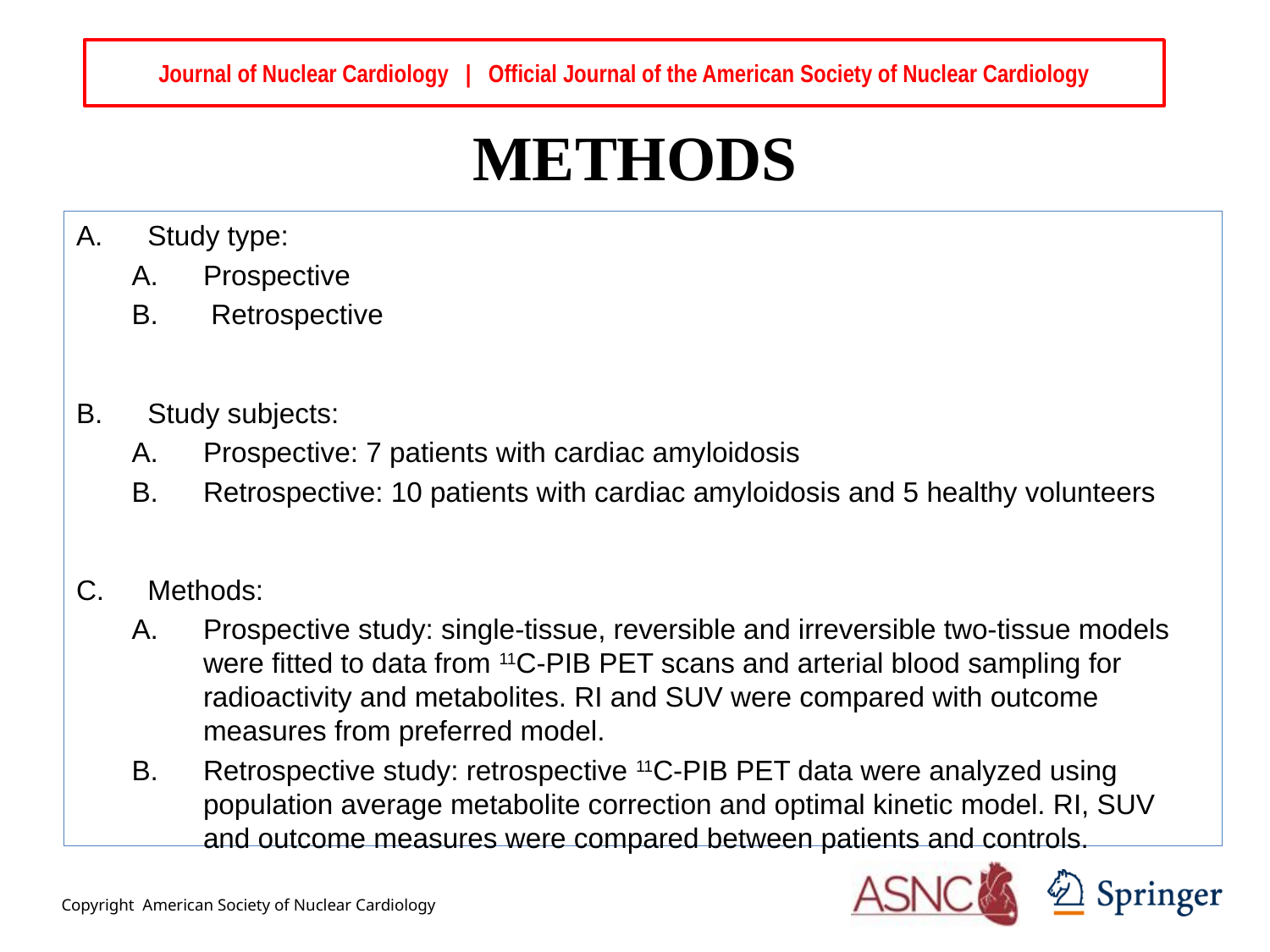

Journal of Nuclear Cardiology | Official Journal of the American Society of Nuclear Cardiology
# METHODS
Study type:
Prospective
 Retrospective
Study subjects:
Prospective: 7 patients with cardiac amyloidosis
Retrospective: 10 patients with cardiac amyloidosis and 5 healthy volunteers
Methods:
Prospective study: single-tissue, reversible and irreversible two-tissue models were fitted to data from 11C-PIB PET scans and arterial blood sampling for radioactivity and metabolites. RI and SUV were compared with outcome measures from preferred model.
Retrospective study: retrospective 11C-PIB PET data were analyzed using population average metabolite correction and optimal kinetic model. RI, SUV and outcome measures were compared between patients and controls.
Copyright American Society of Nuclear Cardiology

## Slide 4
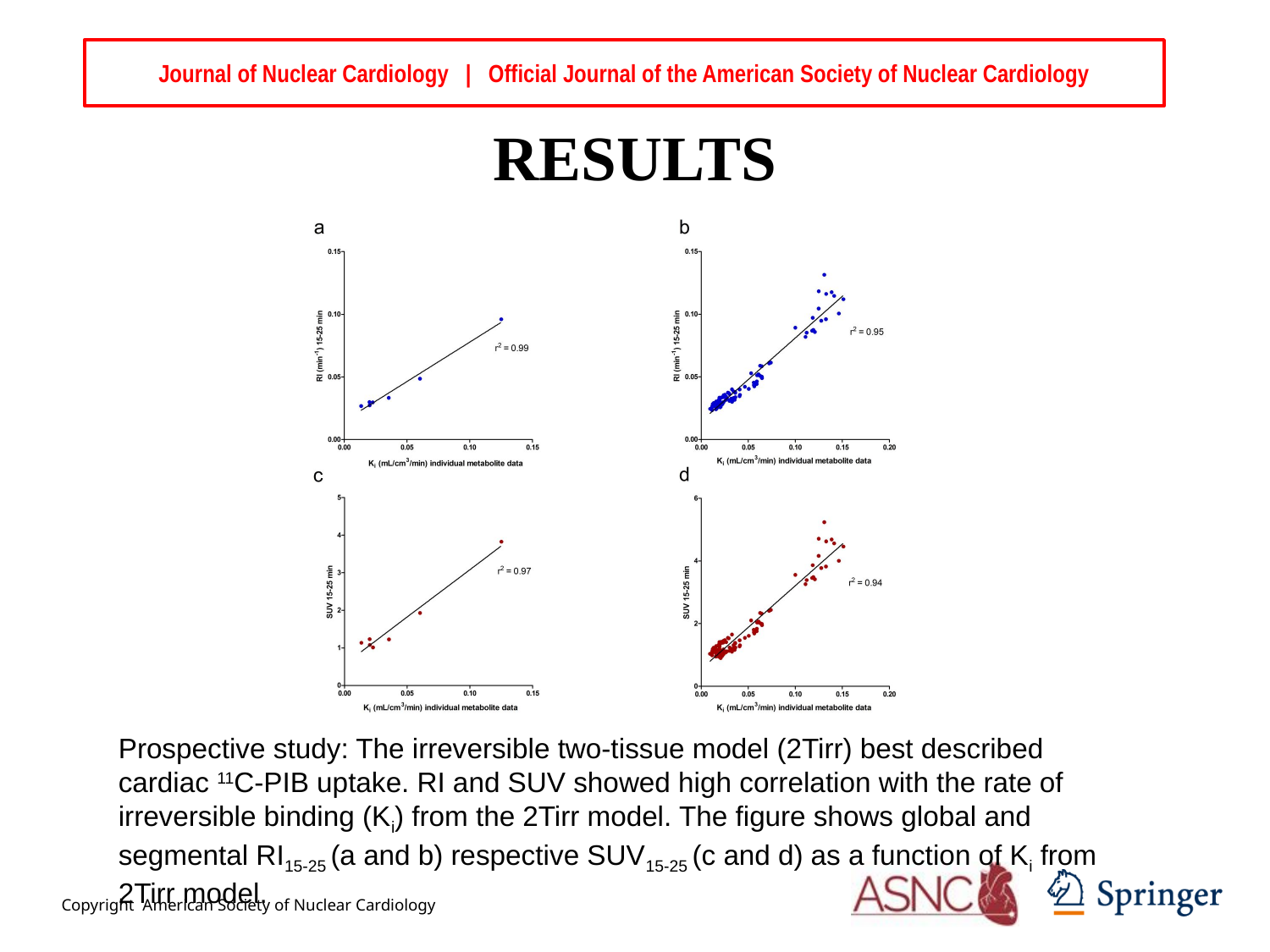

Journal of Nuclear Cardiology | Official Journal of the American Society of Nuclear Cardiology
# RESULTS
Prospective study: The irreversible two-tissue model (2Tirr) best described cardiac 11C-PIB uptake. RI and SUV showed high correlation with the rate of irreversible binding (Ki) from the 2Tirr model. The figure shows global and segmental RI15-25 (a and b) respective SUV15-25 (c and d) as a function of Ki from 2Tirr model.
Copyright American Society of Nuclear Cardiology

## Slide 5
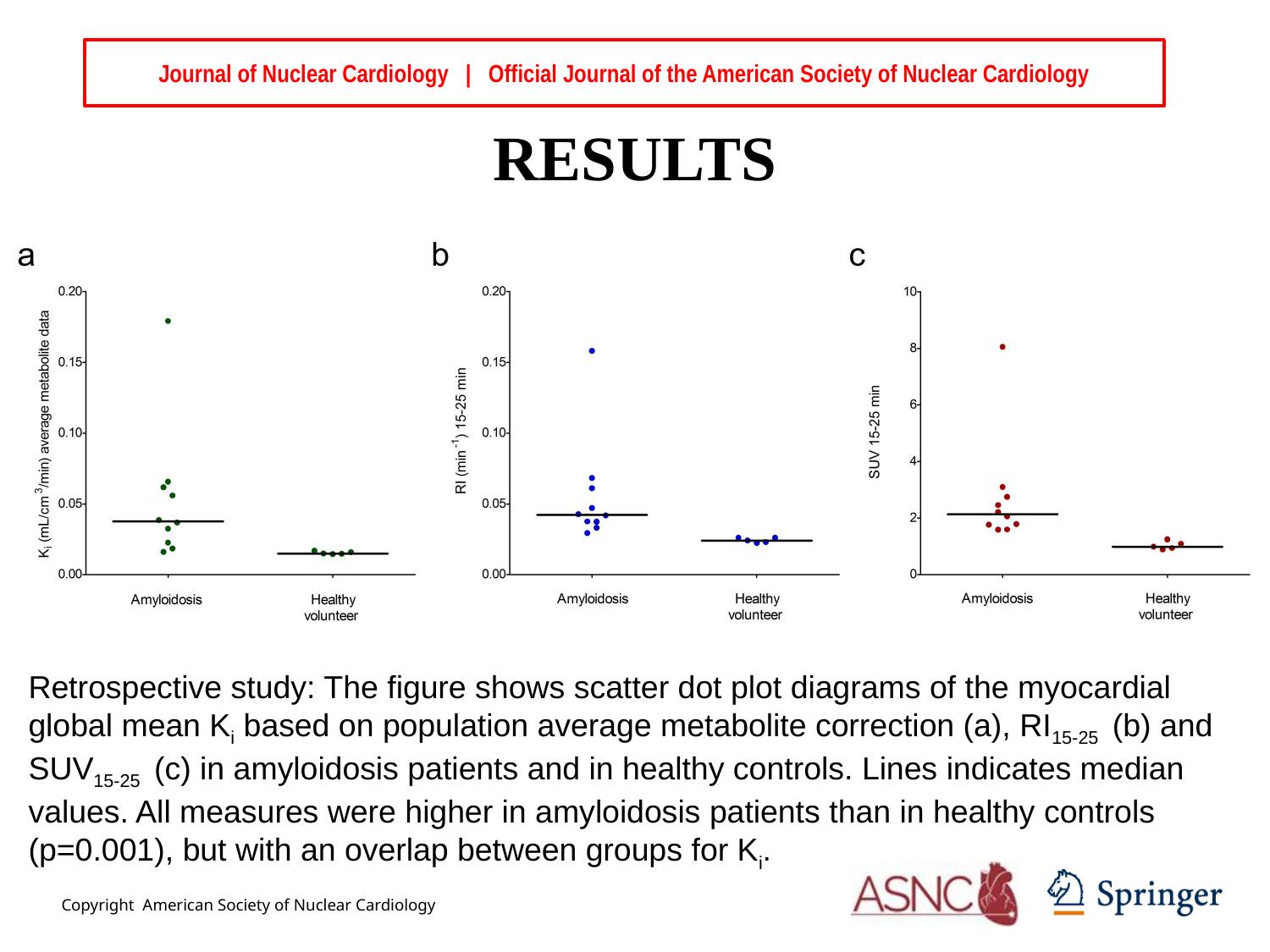

Journal of Nuclear Cardiology | Official Journal of the American Society of Nuclear Cardiology
# RESULTS
Retrospective study: The figure shows scatter dot plot diagrams of the myocardial global mean Ki based on population average metabolite correction (a), RI15-25 (b) and SUV15-25 (c) in amyloidosis patients and in healthy controls. Lines indicates median values. All measures were higher in amyloidosis patients than in healthy controls (p=0.001), but with an overlap between groups for Ki.
Copyright American Society of Nuclear Cardiology

## Slide 6
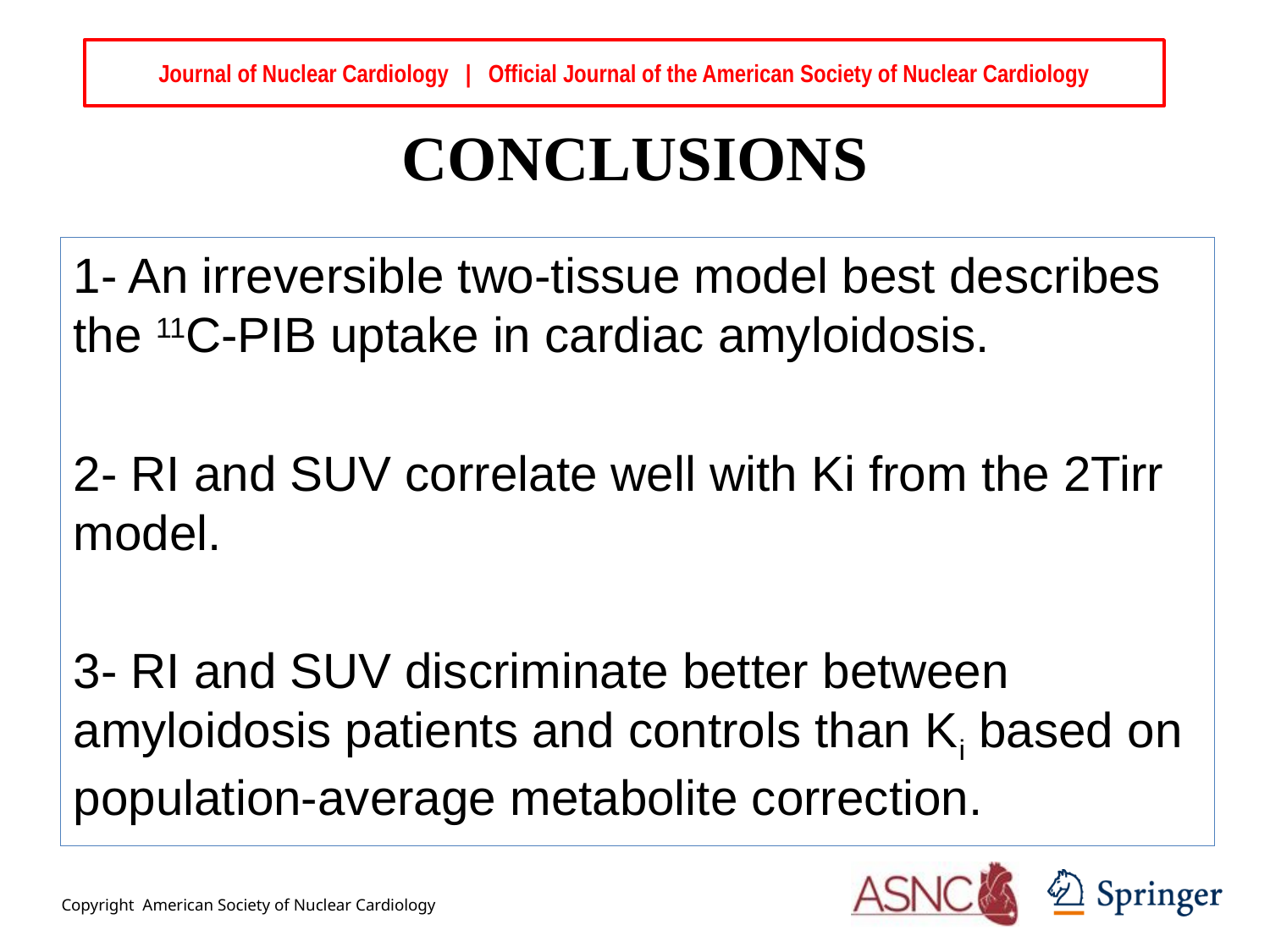

Journal of Nuclear Cardiology | Official Journal of the American Society of Nuclear Cardiology
# CONCLUSIONS
1- An irreversible two-tissue model best describes the 11C-PIB uptake in cardiac amyloidosis.
2- RI and SUV correlate well with Ki from the 2Tirr model.
3- RI and SUV discriminate better between amyloidosis patients and controls than Ki based on population-average metabolite correction.
Copyright American Society of Nuclear Cardiology
